# ARE LEISURE AND CIVIC ACTIVITIES REINFORCING WELL-BEING IN OLDER ADULTS AT RISK OF FRAILTY?

**DOI:** 10.1093/geroni/igz038.2141

**Published:** 2019-11-08

**Authors:** Sarah Dury, Eva Dierckx, liesbeth De Donder, Deborah Lambotte, Daan Duppen, lise switsers

**Affiliations:** 1 Vrije Universiteit Brussel, Brussels, Belgium; 2 Vrije Universiteit Brussel, Brussels, Brussels Hoofdstedelijk Gewest, Belgium

## Abstract

A growing body of work suggest that leisure and civic activities may contribute to the understanding of healthy aging. Yet, only a limited number of studies have examined a less healthy population. Moreover, a broad array of leisure and civic activities tend to be lacking. This paper gives insight into the mechanisms underlying the associations between multidimensional frailty, and well-being with the moderating roles of leisure and civic activities. A two-wave interview survey from the D-SCOPE frailty program was derived using 441 participants aged 60 years and older residing in the Flanders region of Belgium. This study offers evidence that leisure and civic activities buffered the negative relationship between multidimensional frailty and well-being. Moreover, our study identified that for different frailty domains the buffering/moderating role of leisure and civic activities differs in relation to well-being.

